# Bite sized: a mother’s journal alongside anorexia

**DOI:** 10.1186/s40337-015-0045-3

**Published:** 2015-03-14

**Authors:** Susan Ringwood

**Affiliations:** Norfolk, UK

**Keywords:** Anorexia nervosa, Personal experience, Recovery

## Book details

Fiona Hamilton, Vala Publishing Cooperative Ltd, 2014, ISBN: 9781908363114.

## Bite sized

Fiona Hamilton is a therapeutic writing practitioner and mother of three. She is a published poet and has written scripts for the theatre and community projects. In Bite Sized, (168 printed pages), she has made a personal telling of her daughter’s eating disorder in a prose poem. She has captured the very essence of a parent’s experience of having a child with an eating disorder and distilled it into an extraordinarily powerful account. She has taken her hard won insights and through them illustrated the impact on a family when a loved one develops this most challenging of conditions: anorexia nervosa.

Bite Sized is by turns deeply personal, disturbing and ultimately uplifting. Ms Hamilton’s creative gift as a poet has enabled her to craft words with a precision and impact that will resonate with any reader. The structure of the poem follows her experience of struggling to understand her daughter’s illness and of fighting to reclaim her daughter from its grasp.

Those who have their own experience of an eating disorder in their family will find a connection and affirmation in her work. People whose lives have not been touched by anorexia will gain a clear insight of the distorted world around the family and all within it. Clinicians can gain a priceless insight into a family’s turmoil and pain from this prose poem, helping them to be more effective in the support they give to families who are battling an eating in their midst.

The published book Bite Sized makes an impact on its own- the physical presentation of the words on each page shaping the pace and rhythm of the poem’s text. The following quotation is an entire page of the poem:

We brought her meals on a tray

clambering stairs like cumbersome giants

in a shrinking world

Her Dad and I cut up portions

offering bite sized helpings

elfin portions

and sips of water

as if from a thimble

in a desert

On the next page, just the following five lines:

Each tiny portion

weighed a ton

It was backbreaking work

like heaving stones

in the sweltering midday sun

However, Bite Sized is more than the printed page, it is also presented as striking piece of performance art, combining the spoken word of the poem with expressive dance. Bite Sized as a performance will tour schools, health care settings and arts venues in the UK throughout 2015, taking what is ultimately a message of hope driven by the redemptive power of love.

Bite Sized- whether encountered in private reading, the book on your lap that you can’t put down before the last page; or collectively as member of a theatre audience, is an experience that is transformative and unforgettable.

